# A Machine Learning Approach to Predict HIV Viral Load Hotspots in Kenya Using Real-World Data

**DOI:** 10.34133/hds.0019

**Published:** 2023-10-02

**Authors:** Nancy Kagendi, Matilu Mwau

**Affiliations:** Kenya Medical Research Institute, Nairobi, Kenya.

## Abstract

**Background:**

Machine learning models are not in routine use for predicting HIV status. Our objective is to describe the development of a machine learning model to predict HIV viral load (VL) hotspots as an early warning system in Kenya, based on routinely collected data by affiliate entities of the Ministry of Health. Based on World Health Organization’s recommendations, hotspots are health facilities with ≥20% people living with HIV whose VL is not suppressed. Prediction of VL hotspots provides an early warning system to health administrators to optimize treatment and resources distribution.

**Methods:**

A random forest model was built to predict the hotspot status of a health facility in the upcoming month, starting from 2016. Prior to model building, the datasets were cleaned and checked for outliers and multicollinearity at the patient level. The patient-level data were aggregated up to the facility level before model building. We analyzed data from 4 million tests and 4,265 facilities. The dataset at the health facility level was divided into train (75%) and test (25%) datasets.

**Results:**

The model discriminates hotspots from non-hotspots with an accuracy of 78%. The F1 score of the model is 69% and the Brier score is 0.139. In December 2019, our model correctly predicted 434 VL hotspots in addition to the observed 446 VL hotspots.

**Conclusion:**

The hotspot mapping model can be essential to antiretroviral therapy programs. This model can provide support to decision-makers to identify VL hotspots ahead in time using cost-efficient routinely collected data.

## Introduction

Globally, in 2021, 38.4 million people were living with HIV/AIDS, and 650,000 have died of HIV-related illnesses [1]. Even with different lines of successful HIV treatment regimens that are available via global funding, the HIV epidemic is critical in sub-Saharan Africa (SSA), constituting two-thirds of the world’s HIV+ population [2]. Before treatments were available, life expectancy of an HIV-infected person was 10 to 12 years, but with appropriate treatments, it is now possible to live a healthy and full life with HIV.

Sub-Saharan African countries have the highest rates of HIV prevalence. Kenya jointly bears the burden of being the third largest epidemic region, in terms of people living with HIV (PLHIV), along with Uganda and Mozambique [3]. In the past decade, Kenya has made steady progress in combating the HIV epidemic efficiently by implementing progressive disease management policies and procedures. This has resulted in a 44% decrease in new HIV infections from 2010 to 2019 [4]. Kenya is committed to eliminate HIV as a public health threat by the end of 2030 [5]. However, by 2020, the target of reducing new HIV infections by 75% was not achieved [4]. In 2021, 15 counties saw high levels of new infections, thus reversing progress made in the past decade [4]. Missed opportunities to provide HIV testing and treatment services is a primary barrier to a steady decline in new HIV infections.

Kenya has led the way in HIV viral load (VL) monitoring in SSA, with an increase in the number of facilities conducting VL tests since 2016 and an efficient laboratory system [6]. The Kenyan government, together with the help of non-governmental organizations (NGOs) and local community volunteers, is actively trying to curb the spread of HIV by linking patients to health facilities, educating local communities, distributing treatments, and monitoring patients who are on treatment. International organizations such as the United States Agency for International Development and Clinton Health Access Initiative support various initiatives to ensure that PLHIV have access to treatment and care [7]. Kenya’s Ministry of Health strives to improve the VL testing program by strengthening patient tracking mechanisms and VL result utilization. Kenya has a rich and consolidated VL data dashboard that contains routinely collected VL test result information. This dashboard can be leveraged to assess the VL program, follow patient behavior toward medication and treatment, introduce new policies, and identify areas that need improvement. The National AIDS and STI Control Programme (NASCOP), Kenya is a unit within the Ministry of Health that coordinates HIV and AIDS programs in Kenya [8]. Their publicly available dashboard [9] is an analytic platform to follow trends in VL test data and patient outcomes and stores collected data.

Monitoring patients based on CD4 counts and clinical signs often delays proper care, thus increasing the risk of developing antiretroviral drug resistance (DR) [10]. Measurement of HIV-1 RNA levels via VL test is an essential tool to monitor a patient’s HIV status. Per local standard of care, a patient is VL non-suppressed if they have VL of at least 1,000 copies/ml or more after a minimum of 6 months on anti-retroviral with adherence [11]. Regular VL monitoring in patients can reduce morbidity and mortality due to VL non-suppression (VLNS) by optimizing treatment of failing/virally non-suppressed patients. A nation’s approach toward tackling an epidemic is a public health issue at an administrative level (e.g., health facility, subcounty, and county) rather than at individual level. Patient VL data can be aggregated at an administrative level and followed to provide a time-series trend of data.

With the emergence of real-world data, data science, and machine learning (ML) methods in the field of public health, the availability of NASCOP VL data has immense potential yet to be exploited. Identifying hotspots of public health threats has been done by epidemiologists in the past [12,13]. Several studies have explored HIV hotspot identification to quickly detect centers of HIV transmission and implement effective interventions by screening at-risk patients [14]. In a novel approach, we use available VL datasets to predict an HIV hotspot, at a future time point, at an administrative level. Use of routinely collected data is cost-efficient as no extra resources are spent on the collection of data.

ML models have the potential to unlock answers from large and complex datasets [15]. Herein, we describe the use of ML methods to decipher complex associations in our datasets [16,17]. We have developed a niche ML modeling technique to predict HIV hotspots for the upcoming month given the current month’s data. The distribution of hotspots at the county and subcounty level in Kenya could help clinicians as well as policymakers identify the problem areas and take prompt action. Our predictive model could help with planning ahead in time and improving the healthcare facilities, treatment disbursement, patient care, and follow-up uniformly across the entire country.

## Methods

### Data source

This study used 2 data sources related to Kenya’s VL testing and the health facilities that provide these services.

The health facility data are obtained from the website “Kenya Master Health Facility List” (http://kmhfl.health.go.ke/#/home) [18]. This website contains information like geographical location (names of counties and subcounties), administrative location (ward), ownership (name of the private or government entity), regulatory body type, and services offered by all Kenyan health facilities and community units.

We also used pseudonymized laboratory patient-level VL data from NASCOP collected between January 2015 and December 2019. Some predictor variables of interest are demographic characteristics such as age and gender; dates of VL testing, collection of test specimen, dispatch of test results; sample type, antiretroviral regimen, test result, and test justification.

These 2 datasets were linked by key variable(s), health facility name and code, that were common in both data files. The laboratory data contained the names of the facilities that were used as a unique key variable to link the patient-level data with the facility data.

### Data setup

Data cleaning is the first important task in a data science project [19]. The source patient data had a unique identifier, patient ID, to label the pseudonymized patient data. The patient IDs are system-generated codes received with the laboratories dataset, and the authors of this article had no knowledge of their algorithm. Prior to model development, we conducted rigorous data pre-processing. The patient ID data field was refined to rule out data entry errors and repetition of unique IDs with a combination of originally available patient ID, gender, date of birth, and county. All character variables were converted to lowercase, and any leading and trailing spaces were removed and collapsed as one single word. This was particularly helpful to clean special characters from names of facilities and counties. This exercise also ensured proper matching of rows when 2 datasets were being linked with a character variable. The date variables were harmonized to reflect the yyyy-mm-dd format. We attributed dates to the first of the corresponding month if only month and year were present. VL test results had values in various formats, e.g., a numeric entry such as “4”, “1,000”, etc.; “< LDL copies/ml”, “< 150 cp/ml”, etc.; and texts like “Target Not Targeted”, “Sample not received”, “not detected”, “INVALID”, etc. All valid test results were cleaned thoroughly and classified into 3 categories—lower than the detection limit (LDL), low level viremia (LLV), and high viral load (HVL). We attribute a VL test result as LDL if the VL is <400 copies/ml, LLV if it is between 400 and 1,000 copies/ml, and HVL if it is higher than or equal to 1,000 copies/ml.

### Model development

Our modeling data were at a patient and VL test level. Each row was a patient’s laboratory test information at a specific time point. Since our objective is to predict hotspots of HIV VLNS 1 month ahead in time, we aggregated our data at the facility level such that each facility at a specific time was either a hotspot or a non-hotspot. The World Health Organization (WHO) recommends action if 10% of the population reach HIV drug-resistant status [20]. With guidance from HIV subject matter experts, we decided to use 20% as the hotspot threshold for VLNS HIV+ patients. Thus, we defined the outcome, HIV VL hotspot, as facilities with >20% VLNS HIV patients. The outcome variable of hotspot vs. non-hotspot is derived from the VL test result. We combined LLV and LDL outcomes to indicate VLS status while HVL indicated VLNS status of a patient. Thus, VL test results were treated as dichotomous variables where a patient could either be VLS or VLNS. Our dataset had approximately 70% non-hotspot vs. 30% hotspot facilities; indicating a slight imbalance.

The predictor variables used were derived from the laboratory and health facility datasets. The aggregation of data at the facility offered the opportunity for feature engineering predictor variables that may be relevant at the facility level. Since our data were time specific (month–year) at each facility, we summarized the existing variables at this granularity. A few examples of the derived predictor variables are as follows:

1. Number of VL tests and patients per facility/per month–year
2. Number of VL tests and patients per regimen per facility/per month–year
3. Number of regimens per facility/per month–year
4. Number of patients in pre-defined age brackets per facility/per month–year
5. Average and standard deviation of time difference between 2 time points of test logistics (e.g., collected and tested) per facility/per month–year
6. Average, maximum, and minimum times between 2 VL tests for a patient per facility/per month–year

We also created several lagged predictor variables including lagged value of hotspot status in the previous months. Feature engineering of predictor variables alters the feature space by creating new variables that add information and value to the learning process [21]. Since our dataset had a limited number of predictor variables restricted to facility data and laboratory VL tests, deriving new features at the facility level strengthened our model development effort.

Outliers were checked for each predictor variable and treated as missing. Imputation of missing values was done at the facility-level dataset. Character variables were treated as factors and missing values were assigned a separate class. Numeric variables were imputed by replacing missing values with the average of the available value for each variable. We assumed that our data were missing completely at random (MCAR) since missing data implied data entry issues, or lost or damaged samples in the lab. Several imputation methods were considered, but we decided to use mean imputation because it is simple to implement and works for MCAR data [22]. We checked for multicollinearity in the final dataset and dropped predictor variables that were correlated in order to remove redundant information from data.

**Fig. 1.**
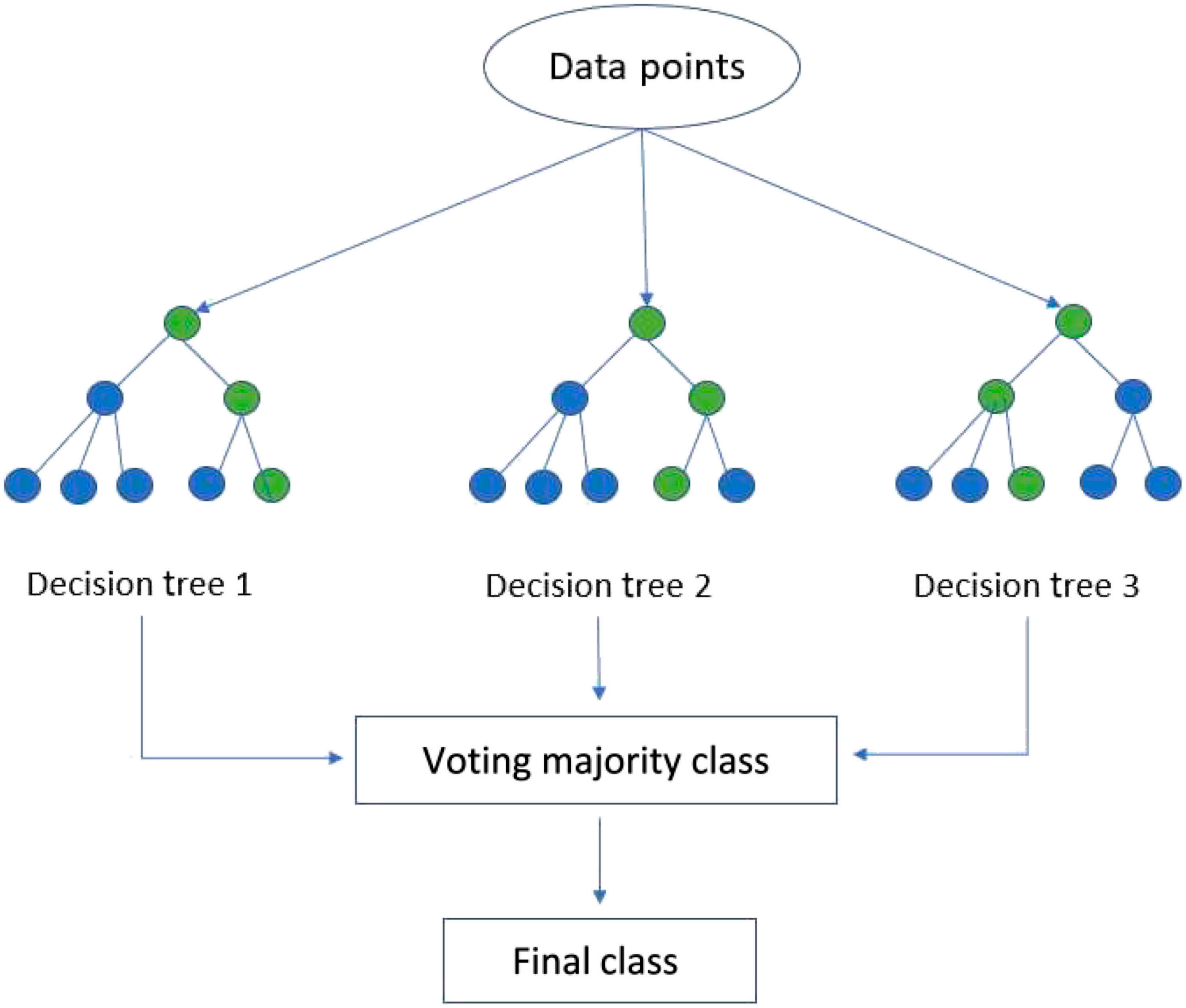
Representation of a simplified random forest model (green and blue dots represent 2 distinct classes).

We developed a random forest [23] model to predict HIV hotspots in Kenya, 1 month ahead in time, via a supervised learning approach. The outcome variable was hotspot status of a health facility and predictor variables were derived from the laboratory and facility datasets. A random forest classification model is an ensemble of several decision trees that classifies by voting for the most popular class (Fig. 1).

The model dataset was partitioned into train and test datasets in a 75–25 split. Of the original data, 25% was set aside to test the proposed model. Note that, since our target was imbalanced, we split the data based on the outcome variable such that the imbalance proportion was retained in both train and test datasets. Due to data imbalance, reporting just model accuracy is not appropriate. We introduce several other performance metrics that validate model performance for imbalanced data.

Sensitivity is the proportion of truly identified hotspots (true positive) among all actual hotspots (positive) and precision is the model’s ability to predict a true hotspot (true positive) as opposed to predicting a non-hotspot as hotspot (false positive) (Table 1).

**Table 1.** Confusion matrix prototype.

		Predicted	
		Hotspot	Non-hotspot
**Actual**	**Hotspot**	True positive (TP)	False negative (FN) (type II error or missed opportunity)
	**Non-hotspot**	False positive (FP) (type I error or false alarm)	True negative (TN)

Typically, we aim to reduce both type I and type II errors (Table 1), but with a fixed sample size, minimizing both errors simultaneously is not always feasible [24]. Thus, we try to minimize the error that would have serious repercussions if not controlled. In this setting, missing a hotspot could adversely impact treatment behavior and delivery of many vulnerable patients. Our motivation was, thus, to reduce the type II error (Sensitivity = 1 – type II error) or increase the proportion of truly identified hotspots among all hotspots. On the other hand, we also did not want our model to produce too many false alarms, i.e., reduce the model precision. Too many false alarms can cause wastage of resources and cripple administrative policies in hours of need, especially in low- and middle-income countries (LMICs). Thus, we chose a threshold value of predicted score that optimizes the trade-off between sensitivity and precision.

The Brier score was a measure developed to scale the accuracy of weather forecasts based on Euclidean distance between the actual outcome and the predicted probability assigned to the outcome for each observation [25]. The score ranges between 0 and 1 with lower scores being desirable. Other common model metrics like specificity, F1 score, area under the curve (AUC) of receiving operating characteristic (ROC), and precision–recall (PR) curves were also reported. The F1 score is a harmonic mean of sensitivity and precision metrics. The higher the value (ranges between 0 and 100%) of AUC on the ROC and PR curves, the better is the model fit.

We have, further, explored model performance using data beyond 2019. We used laboratory and health facility data from January 2020 to March 2022. We wanted to perform a validation with completely out-of-sample data to test the robustness of our model. All model performance metrics were derived based on this dataset along with data characteristics visualizations.

## Results

### Data characteristics

The VL data from 2015 to 2019 had more than 4 million test records. The tests increased over time—while 176,415 tests were performed in 2015, around 1.5 million tests were performed in 2019. This was a result of the increase in number of facilities joining NASCOP, improved test turnaround time of the VL testing laboratories, and scaled up patient outreach for VL tests. The data repository consisted of VL test records of over 2 million patients generated in 4,265 health facilities, of which 3,707 are uniquely linked to patients. Routine VL tests were usually done annually, but a VL test could be done at any time prior to the annual routine test if a patient’s health deteriorated or if there was a life-changing event like pregnancy.

**Fig. 2.**
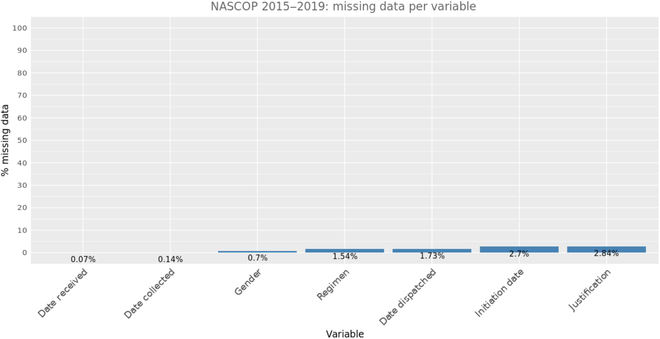
Missing data per variable among original input variables. Age, county, subcounty, facility code, received status, treatment delivery organization name, sample type, testing lab name, and result do not have any missing data.

**Fig. 3.**
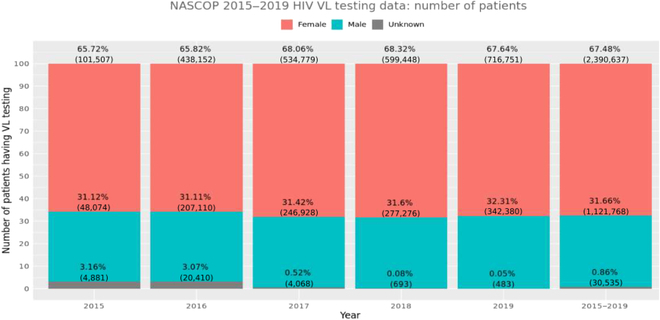
Distribution of patients who had a VL test by gender.

Only 7 variables in our dataset had the issue of missingness (Fig. 2). There are more female patients (68%) tested than male patients due to their health-seeking behavior during childbearing ages; their distribution over the years is captured in Fig. 3.

**Fig. 4.**
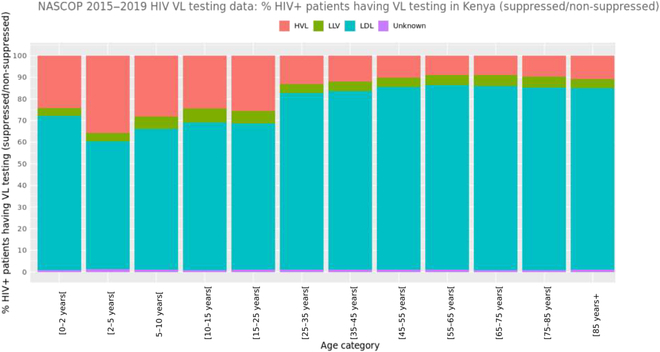
Distribution of HIV+ patients who had a VL test by age and result categories.

In every age group, 60% to 80% HIV+ patients were virologically suppressed at the time of their test (Fig. 4); nevertheless, more than 20% of the patients were non-suppressed, with at least 1,000 copies/ml, in the younger age categories (i.e., <25 years).

The majority of VL tests were done as a routine (Table 2). About 3.56% of tests did not have a valid justification entry in the dataset and single drug substitution justified around 2.75% of all VL tests done.

**Table 2.** Distribution of VL test justification.

Justification	%
Routine VL	86
Confirmation of treatment failure (repeat VL)	4.97
Unknown	3.56
Single drug substitution	2.75
Baseline	1.13
Other	0.71
Clinical failure	0.33
Pregnant mother	0.26
Breastfeeding mothers	0.24
Confirmation of persistent low-level viremia (PLLV)	0.04

### Model validation and performance

With the help of subject matter experts and correlation analysis, we selected 68 variables out of 1,008 derived variables to be included in the model. The random forest model for classifying hotspot against non-hotspots in the upcoming month was applied on the test dataset to validate its performance. We achieved an accuracy of 78% on the test dataset. The sensitivity is 70% and the specificity is 83% (Table 3).

**Table 3.** Performance metrics on test data.

Metric	Value
Accuracy	78%
Sensitivity (recall)	70%
Specificity	83%
Precision	68%
F1 score	69%
Brier score	0.139

**Fig. 5.**
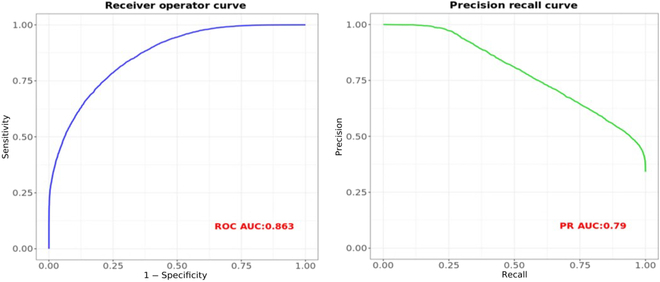
Left panel: ROC curve; right panel: PR curve on test data.

Our model had an F1 score of 69% with 68% precision. The AUC of ROC curve was 86% and PR AUC was 79% (Fig. 5) and had a Brier score of 0.139.

While VL results in prior months were the 2 most important variables, feature engineered variables with time of treatment initiation, testing, test collection, test dispatch to laboratories, and test receipt times were some of the important variables. We also saw some factors related to the health facilities like county, ward, regulatory body, and subcounty feature as important variables in the model. The variable importance bar plot is added as a Supplementary Material (Fig. S1).

### Hotspot visualization

**Fig. 6.**
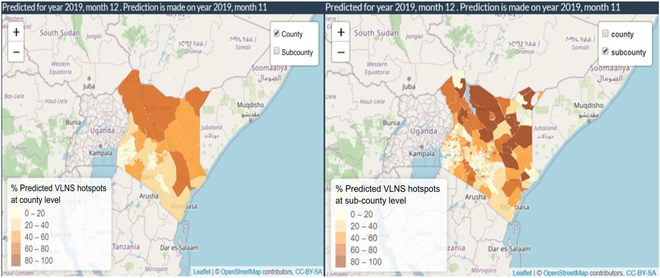
Percentage VLNS hotspot mapping at Kenya county level (left) and subcounty level (right).

Predicted HIV hotspot distribution at the county and subcounty levels for 2019 December is included (Fig. 6); the prediction was made in 2019 November. The color ranges from light yellow to brown; light yellow means lower percent of hotspot and brown means higher percent of hotspots. % Hotspots were calculated based on all facilities present in that area (as per our dataset).

Although the southwest bay area has high prevalence of HIV, HIV monitoring programs are efficiently deployed here, making this region a non-hotspot area. On the other hand, the northern parts of Kenya are hard to access due to a lower population density in regions with a high risk of conflict; thus, facilities in these areas have a higher occurrence of hotspot status.

**Fig. 7.**
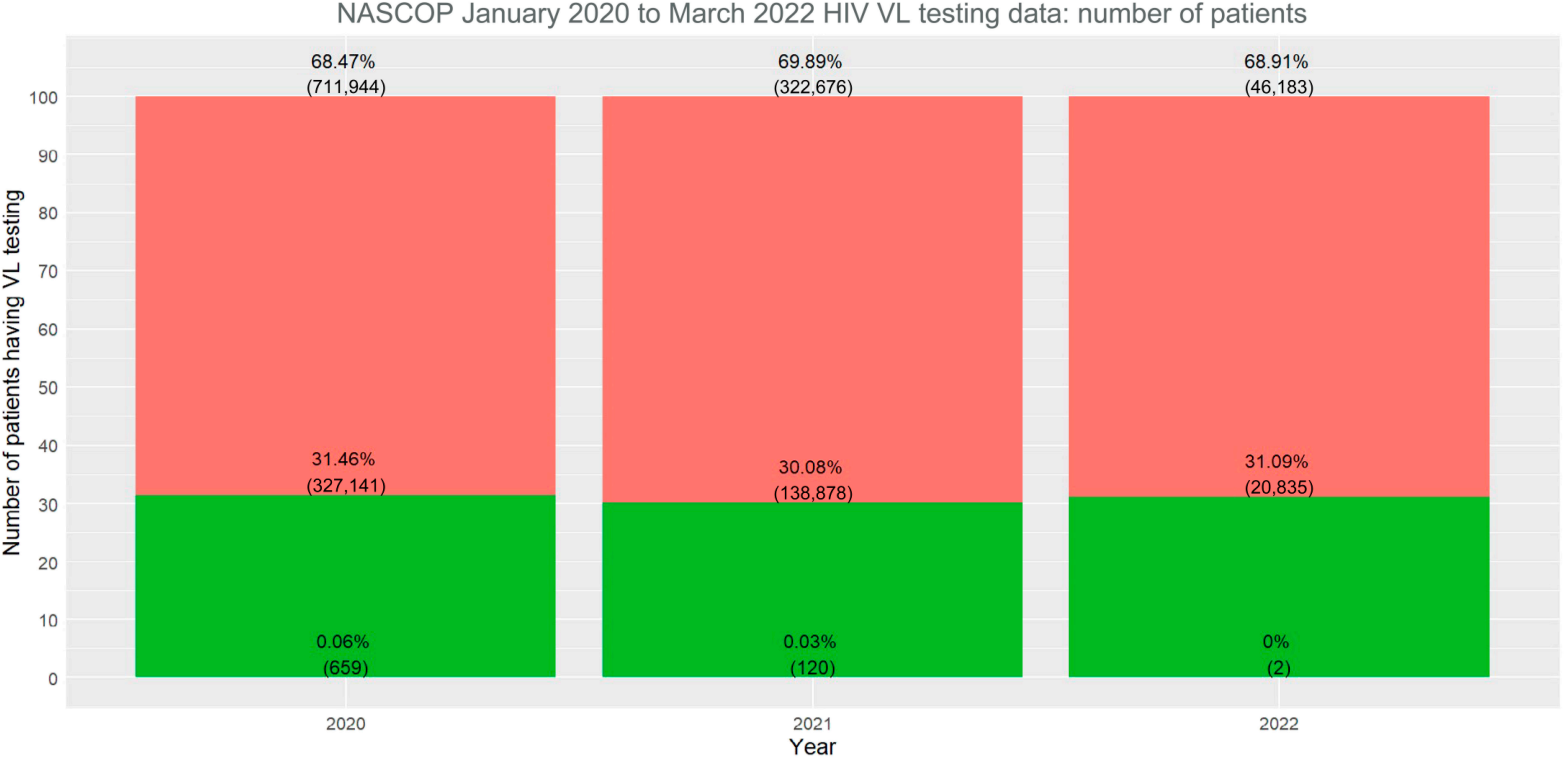
Distribution of patients who had a VL test by gender (January 2020 to March 2020 data).

**Fig. 8.**
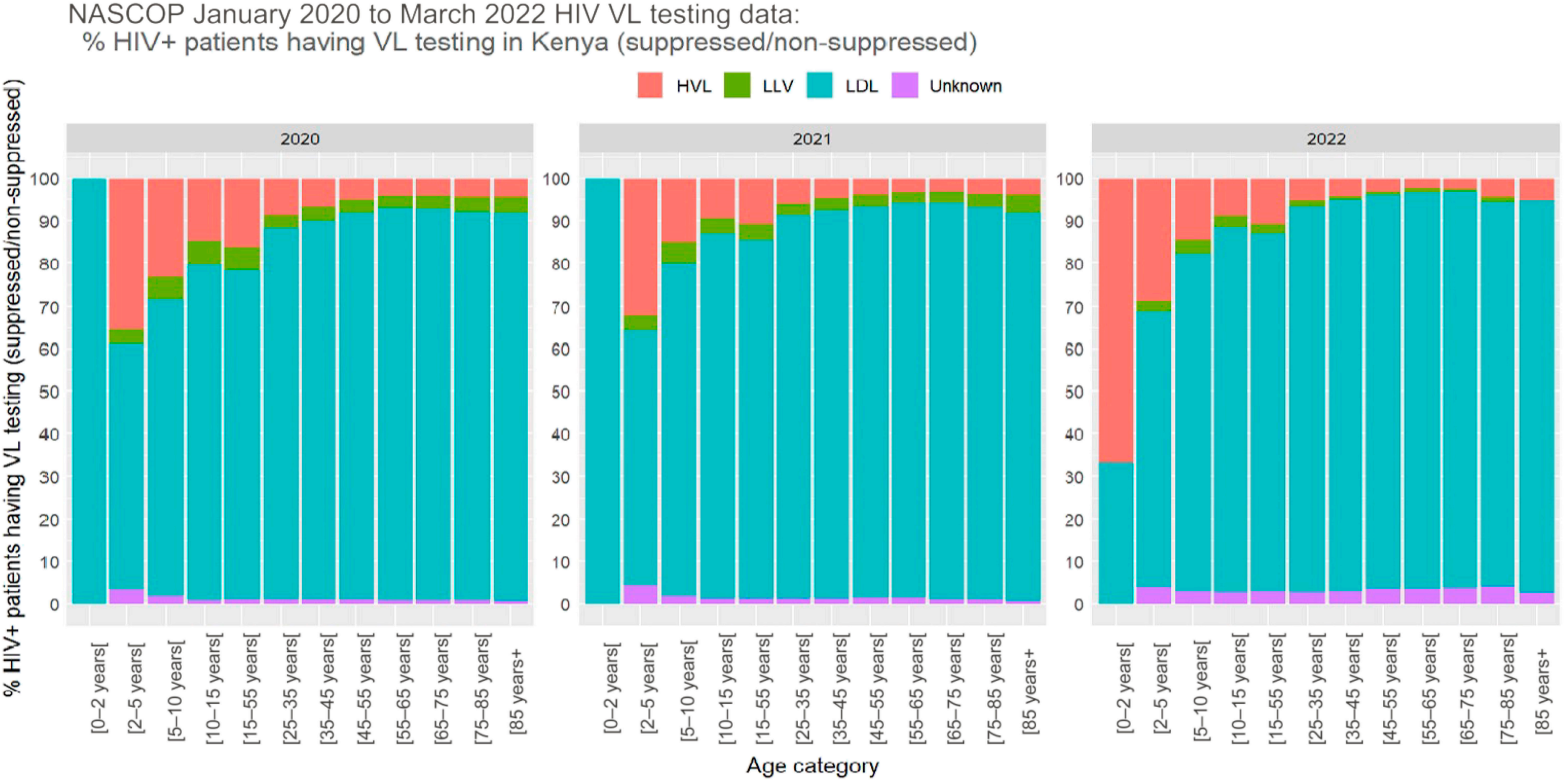
Distribution of HIV+ patients who had a VL test by age and result categories (January 2020 to March 2020 data).

In addition to the test dataset aside from training data, we validated our random forest model based on completely new data. We retrieved laboratory and health facility data from January 2020 to March 2022. These data were used to validate the model as a complete out-of-the sample model validation. The data characteristics are visualized in Figs. 7 and 8.

The overall data distributions had not changed substantially over the years from 2015 to 2022. Note that 2022 only had data up to March, thus skewing some variable distributions.

The results from this validation are provided in Table 4.

**Table 4.** Performance metrics on out-of-sample validation data.

Metric	Value
Accuracy	79%
Sensitivity (recall)	66%
Specificity	82%
Precision	40%
F1 score	50%
Brier score	0.10

**Fig. 9.**
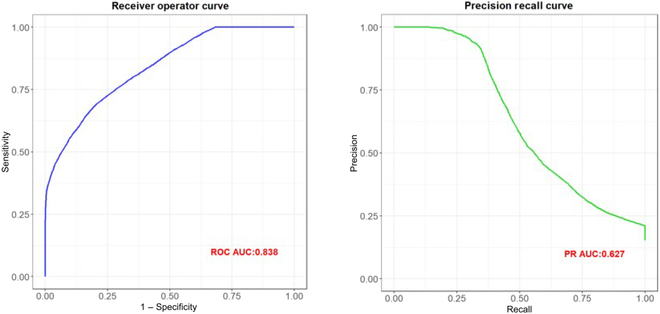
Left panel: ROC curve; right panel: PR curve on test data (January 2020 to March 2020 data).

The model performance on January 2020 to March 2022 data showed comparable results to the 2015 to 2019 data validation results. From the 83% ROC AUC (Fig. 9), it was evident that the model was a good fit for the new data. We saw that the precision had decreased when compared to the previous validation data, indicating that the new data predictions had higher false positives.

## Discussion

Although Kenya has made considerable progress in its fight against HIV, VL hotspot prediction at a granular geographical location level in high-burden Kenya as an early warning system has not been explored previously. To the best of our knowledge, this is a novel approach of mapping out the heavily HIV-affected regions within the country ahead in time. Researchers in Kenya have enthusiastically explored the idea of using data and data science [26,27] for HIV surveillance, but the efforts have been limited to small scale or at an exploratory level only. Given the availability of large datasets from routinely collected VL test results and health facility data, the potential of leveraging this information to break down complex administrative and treatment behavior situations is huge. In an effort toward capacity building in data science, the partnership between Janssen and KEMRI entailed recruiting a young female Master of Science researcher to transfer the research learning and expertise.

Kenya’s data are rich and informative, and a preliminary data analysis demonstrated important patterns in the data. We used the available data to find general trends of various feature variables and their association with VL testing. This established the usefulness of the data sources and validated data quality. The skewed gender distribution in the data may imply that men are in general reluctant to get tested. There is a prevention of mother-to-child transmission of HIV program in Kenya, which could also lead to the observation of more females in the dataset. This suggests the need of increased awareness among male patients about the national VL testing program and its benefits. The distribution of HIV+ patients by age and result categories indicates the gaps that still exist in effective treatment outreach among younger patients (Fig. 4).

The random forest classifier developed in this article classifies HIV VL hotspots for the upcoming month with high sensitivity and precision. The advantages of random forest models are that they are robust to outliers and noise, faster than bagging or boosting, simple to implement, and easily parallelized. The AUCs of the ROC and PR curves further establish its high performance. The classifier model covers the entire length and breadth of the country to identify the regions that need closer attention. The second most prevalent reason for conducting a VL test was confirmation of treatment failure (Table 2). This implies that tests are done once a patient reports treatment failure. Our early warning system can detect these high-risk patients before they report treatment failure.

There is no WHO-recommended threshold for VLNS hotspot. WHO recommends 10% threshold for HIV DR hotspot. We discussed with HIV subject matter experts, reviewed literature, and looked at the data at hand to choose 20% as the threshold for VLNS hotspot. Our data have around 20% patients who are virologically non-suppressed. According to a thorough literature review, we found that VLNS in LMICs range between 15% and 30% [28,29]. Not all VLNS patients will develop DR, but since 15% to 30% of HIV patients are VLNS in LMICs and 10% is the WHO-recommended DR hotspot, we decided to choose a number that falls within the range of VLNS status but is higher the DR threshold.

Despite many advantages of our approach in using big data and data science to predict HIV hotspots in Kenya, the study has some limitations. Unavailability of comprehensive datasets is an outstanding problem in LMICs. The data available comprise VL test results of patients and health facility characteristics. Although socio-economic information such as household income, education level, marital status, and employment have been associated with the risk of HIV infection [30], such data are unavailable, or data sources cannot be linked. DR in HIV could be explored, although not all HIV patients in Kenya are eligible for a DR test. Sustainability and maintaining the learnings at KEMRI and for the country in Kenya to ensure that the scope of this research brings value in management of PLHIV is important. Finally, the consistent usability of the outcome to address and fill the gaps and explore generalizability of our model in other settings (outside of Kenya) will be explored in future studies.

The data used for modeling was from 2015 to 2019. We used 75% of these data to train the random forest model and the remaining to validate the model. Additionally, we used a separate dataset from January 2020 to March 2022 to validate the model performance. With the new data, the model had similar accuracy with a comparable rate of identifying true positives but higher rate of false positives. Since we aim to find the best trade-off between type I and type II errors (Table 1), the slightly higher false positives are acceptable. From a policymaking perspective, the need to identify true hotspots are of higher importance as compared to having a few non-hotspots identified as hotspots. Nevertheless, this highlights the importance of validation and continuous monitoring of model performance with new data [31]. With time, the distribution of a population shifts, and this can cause a model to digress from its expected behavior. We recommend that the model be updated with newer data from time to time to reflect the present conditions of the concerned population.

## Conclusion

Kenya has used administrative level data insights to implement policy changes for HIV health prevention and treatment [32]. Our study shows that the use of real-world data and ML methods to build an early warning model can support LMICs in their decision-making processes, including some geographical characteristics of the facility at a specific time point.

The internal validation using new routinely collected data highlighted the necessity of surveillance of ML models and retraining them as and when needed. ML models are a great asset in the age of technology—to help healthcare workers, policymakers, and administrative leaders to make data-driven decisions. However, we should also be cognizant that ML models’ outputs will change if there is a population shift. For example, when an LMIC makes gradual development in its many sectors, thus affecting a shift in the population demography, ML models should be retrained to capture this shift. The random forest model we built as an early warning system is robust since it has consistent accuracy and AUC ROC across data from different time points, but it needs continuous monitoring to check for any anomaly in the predictions.

Early identification of health facilities that report more than 20% of patients with VLNS status will not only ensure improvement in treatment delivery, tests conducted, and patient outreach and retention but also help in focusing on locations that may be regions of conflict or prone to drug-resistant HIV [33,34]. VLNS status could be related to challenges associated with distribution of treatments, availability of medications, poor infrastructure, or limited accessibility by patients due to poor road connectivity. Actionable interventions to improve these conditions will benefit Kenya in holding the reigns of increasing VLNS and DR. Many researchers have delved into the threat of HIV DR in Kenya [35,36]. However, little work has been done to actively identify poorly performing locations to tackle HIV DR. Our fast and effective method based on routinely collected data can identify poorly performing locations and accelerate the administrative processes in managing VLNS and HIV DR.

### Ethical approval

Permission for the study was granted by the Scientific Ethical Review Unit (SERU) of the Kenya Medical Research Institute (KEMRI), approval KEMRI/SERU/CIPDCR/040/3970. This study does not access human participants’ personal information data and does not involve patient samples. It accesses data available publicly that have pseudonymized participant identifiers. For that reason, consent forms are not used. The data use poses minimal confidentiality issues or risks.

## Data Availability

De-identified, individual participant data, a data dictionary defining each field in the dataset, will be made available to others after publication of this manuscript, following approval of a proposal. Proposals should be directed to mmwau@kemri.go.ke. To gain access, data requestors will need to sign a data access agreement. Requests for model scripts and a synthetic dataset should be directed to mmwau@kemri.go.ke.

## Supplementary Materials

Supplementary 1 Fig. S1. Bar plot of variable importance of the random forest model.
