# Supplementary figures and images for: A Machine Learning Approach to Predict HIV Viral Load Hotspots in Kenya Using Real-World Data
Source: Health Data Sci. 2023 Oct 2;3:0019. doi: 10.34133/hds.0019 (PMC10880164; doi:10.34133/hds.0019)

Figure S1

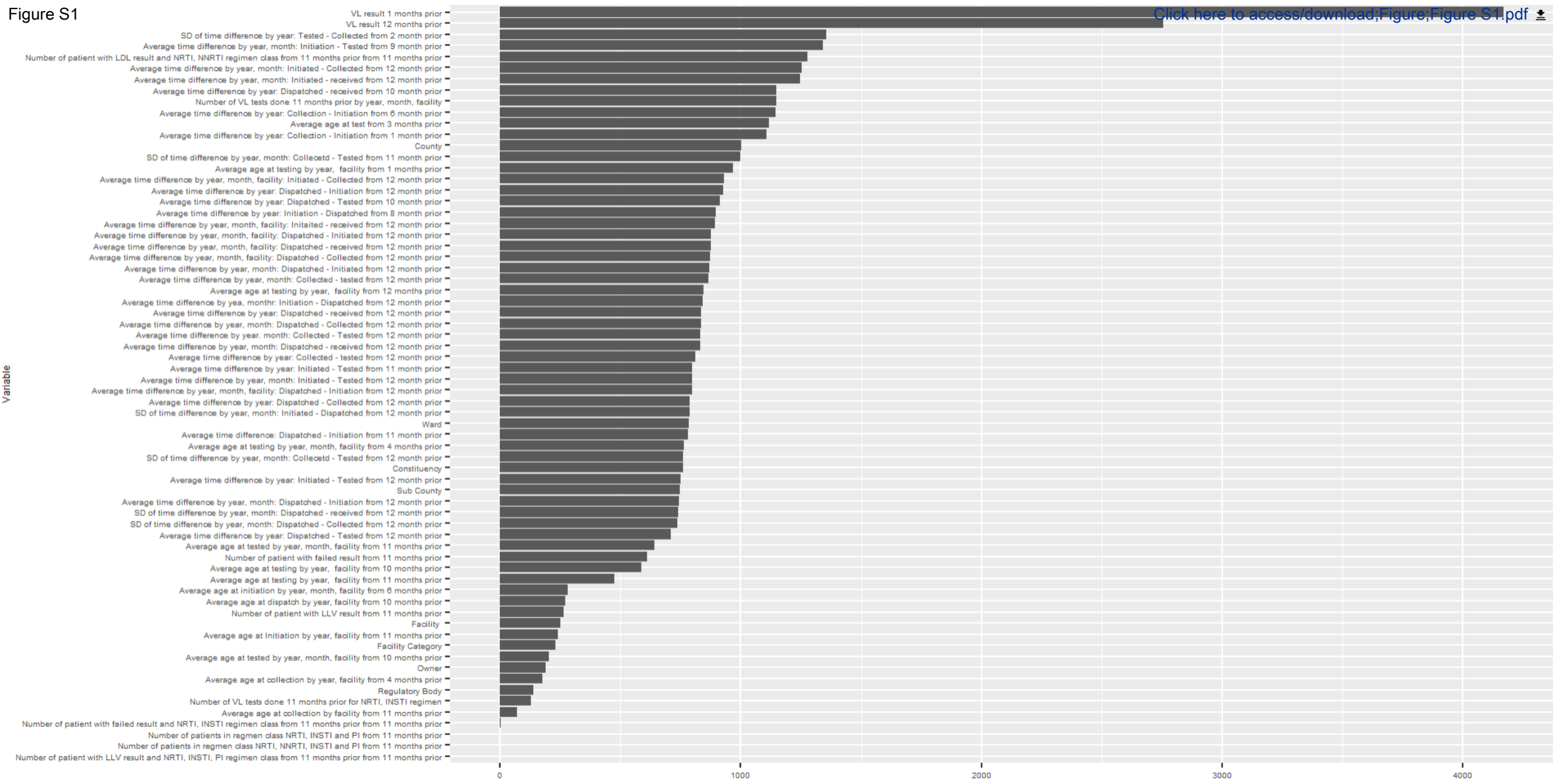

Supplement: Supplementary 1 — Fig. S1. Bar plot of variable importance of the random forest model. [file hds.0019.f1.zip › FigS1.pdf]
